# Carbon Nanohorns Carried Iron Fluoride Nanocomposite with ultrahigh rate lithium ion storage properties

**DOI:** 10.1038/srep12154

**Published:** 2015-07-15

**Authors:** Lishuang Fan, Bingjiang Li, Naiqing Zhang, Kening Sun

**Affiliations:** 1 Department of Chemistry, Harbin Institute of Technology, Harbin, China; 2 State Key Laboratory of Urban Water Resource and Environment, Harbin Institute of Technology, Harbin, China; 3 Academy of Fundamental and Interdisciplinary Sciences, Harbin Institute of Technology, Harbin, China

## Abstract

Novel hierarchical carbon nanohorns (CNHs) carried iron fluoride nanocomposites have been constructed by direct growth of FeF_3_·0.33H_2_O nanoparticles on CNHs. In the FeF_3_·0.33H_2_O@CNHs nanocomposite, the mesopore CNHs play the role as conductive matrix and robust carrier to support the FeF_3_·0.33H_2_O nanoparticles. The intimate conductive contact between the two components can build up an express way of electron transfer for rapid Li^+^ insertion/extraction. The CNHs can not only suppress the growth and agglomeration of FeF_3_·0.33H_2_O during the crystallization process, but also sever as an “elastic confinement” to support FeF_3_·0.33H_2_O. As was to be expected, the hierarchical FeF_3_·0.33H_2_O@CNHs nanocomposite exhibits impressive rate capability and excellent cycle performance. Markedly, the nanocomposite proves stable, ultrahigh rate lithium ion storage properties of 81 mAh g^−1^ at charge/discharge rate of 50 C (a discharge/charge process only takes 72 s). The integration of high electron conductivity, confined nano sized FeF_3_·0.33H_2_O (~5 nm), hierarchical mesopores CNHs and the “elastic confinement” support, the FeF_3_·0.33H_2_O@CNHs nanocomposite demonstrates excellent ultrahigh rate capability and good cycling properties.

Lithium-ion battery (LIB), as the main energy storage device, captures the portable electronic device market due to their high energy density, high output voltage, long span life and environmental benignity^1^,^2^. In order to master the upcoming markets of large scale electrochemical energy storage, electric vehicle and hybrid electric vehicles, great improvements in electrochemical performance are eagerly required^3^,^4^. The phase of market is shifting from the early stage to the next stage as demands for higher energy and power densities are growing^5^,^6^. To improve the electrochemical performance of LIB further to meet the requirement of all-electric vehicles and grid energy storage, alternative cathodes with a significant reduction in cost and great improvement in capacity and power density are urgently needed^7^,^8^,^9^,^10^,^11^,^12^.

As a typical representative of the late-model LIB cathode material, iron fluoride has attracted rapidly increasing amount of attention due to its high theoretical capacity, abundant sources, and relatively low cost^13^,^14^,^15^,^16^,^17^,^18^. Among the numerous polymorphs of iron fluoride (such as FeF_3_, FeF_3_·0.33H_2_O), FeF_3_·0.33H_2_O is the most studied crystal form due to its unique tunnel structure which is greatly beneficial for the Li^+^ storage performance^19^. However, its implementation in LIBs is greatly hindered by its poor power performance^15^,^16^,^20^. To further improve the power density of the FeF_3_·0.33H_2_O, various endeavors have been made to overcome the draw backs by hybridizing it with a conductive additive phase (such as CNTs, graphene and ordered mesoporous carbon)^21^,^22^,^23^,^24^, which provides a facile electron pathway.

As a number of nanostructured carbon materials (such as CNT^25^, carbon nanohorns (CNHs)^26^), CNHs have received an ever increasing attention^27^,^28^,^29^. CNHs have large surface area, hierarchical mesoporous structure, excellent electrical conductivity and a similar tubular structure to single-walled carbon nanotubes. Due to these superiorities on structure, CNHs have been widely studied for various applications, such as catalyst support^30^, electrochemistry bio-sensing^31^, supercapacitor^32^, fuel cells, and so on^33^. Thus, CNHs hold promise as a good conductive matrix to support iron fluoride for enhancing the electrochemical performances as LIBs cathode materials.

In this work, a facile and advanced architecture design of carbon nanohorns carried FeF_3_·0.33H_2_O nanocomposites (FeF_3_·0.33H_2_O@CNHs) is presented for the first time. In this composite electrode, the CNHs not only build up the conductive matrix that provides a charge transfer express way to FeF_3_·0.33H_2_O, but also works as an “elastic confinement” carrier to disperse and support the FeF_3_·0.33H_2_O nanoparticles. Moreover, the hierarchical, mesoporous channels provide a large specific surface area which can effectively increase the electrolyte-electrode interface, assuring that the electrolyte can sufficiently filtrate into the electrode materials. As was expected, at a charge/discharge rate of 1 C, FeF_3_·0.33H_2_O@CNHs nanocomposite can deliver a stable specific capacity of 154 mAh g^−1^ after 50 cycles with capacity retention over 95%. Remarkably, the nanocomposites exhibit stable lithium ion storage properties of 81 mAh g^−1^ even at an ultrahigh charge/discharge rate of 50 C (10 A g^−1^, 72 s for complete charge/discharge). To the best of our knowledge, such impressive superior power rate for iron-fluoride based electrodes has not been reported previously.

## Results and Discussion

**Figure 1:**
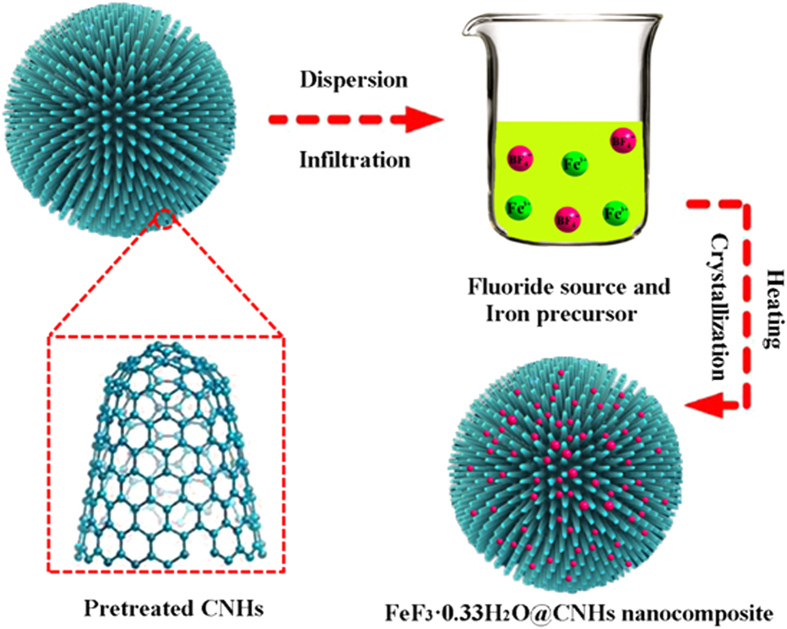
The synthesis procedure of the FeF_3_·0.33H_2_O@CNHs nanocomposite.

In Fig. 1, the schematic diagram of synthesis process of FeF_3_·0.33H_2_O@CNHs nanocomposite is shown. The pre-treated CNHs were fully dispersed in alcohol by powerful ultrasonic dispersion. Then the iron precursor and fluoride source were successively added into the solution. To ensure complete infiltration of the CNHs, thoroughly agitation was carried out by stirring under the protection of nitrogen. The crystallization procedure proceeded when the reaction system was heated. During the crystallization process, the CNHs work as the conductive carrier to support the FeF_3_·0.33H_2_O nanoparticles.

**Figure 2:**
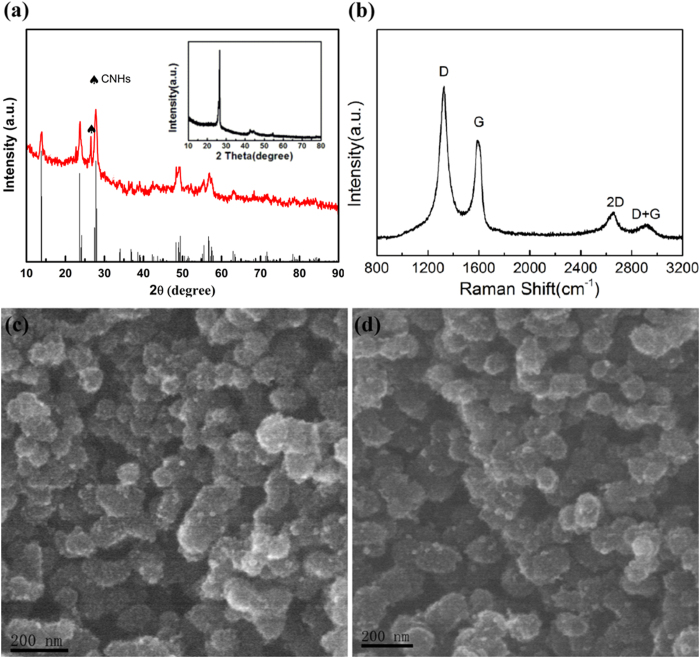
(**a**) The X-ray diffraction of the FeF_3_·0.33H_2_O@CNHs nanocomposite, the inset of (**a**) is the XRD of the CNHs, (**b**) Raman spectra of the FeF_3_·0.33H_2_O@CNHs nanocomposite, (**c**) The SEM image of the pre-treated CNHs and (**d**) FeF_3_·0.33H_2_O@CNHs.

X-ray diffraction (XRD) experiments were carried out to get the structure and composition of the FeF_3_·0.33H_2_O@CNHs nanocomposite. As shown in Fig. 2a, most typical peaks of HTB-FeF_3_·0.33H_2_O (JCPDS 76-1262)^15^,^19^,^34^,^35^ could be identified in this composite and is different for FeF_3_·0.33H_2_O (JCPDS 026-0783) shown in the Fig. S1. Meanwhile, the result of the TG analysis is similar with the previous report^19^,^36^. These results are proved to be the formation of FeF_3_·0.33H_2_O and good stability of the FeF_3_·0.33H_2_O (Fig. S2).The additional peak observed around 26° is resulted from CNHs (inset of Fig. 2a). From the elemental analysis, the carbon element content is about 16 wt % in the nanocomposite. The presence of CNHs in the composite is confirmed by Raman spectroscopy as shown in Fig. 2b.

D-band line (ca. 1326 cm^−1^) and the G-band line (ca. 1591 cm^−1^), respectively. The The Raman spectra of FeF_3_·0.33H_2_O@CNHs exhibit the regular two peaks, corresponding to the D-band is caused by edges or structural defects which can break the selection rule and symmetry; the G-band is related to the vibration mode in graphite-like carbon^26^,^37^. The 2D band (ca. 2663 cm^−1^) confirms the multilayer structure of the graphite. CNHs with defects would show an additional disorder related peak, broadening (D + G) band at 2920 cm^−1^, originating from a combination mode.

The structure and morphology of CNHs and the nanocomposites were investigated by scanning electron microscopy (SEM). In Fig. 2c, the typical SEM image of the CNHs shows a spherical bundle structure with a rough surface. Protrusions of several nanometers in size are observed on the spherical bundle surface due to individual nanohorns growing from the center of the bundle. After FeF_3_·0.33H_2_O loading (shown in Fig. 2d), the morphological change in the SEM image of the FeF_3_·0.33H_2_O@CNHs is not notable.

**Figure 3:**
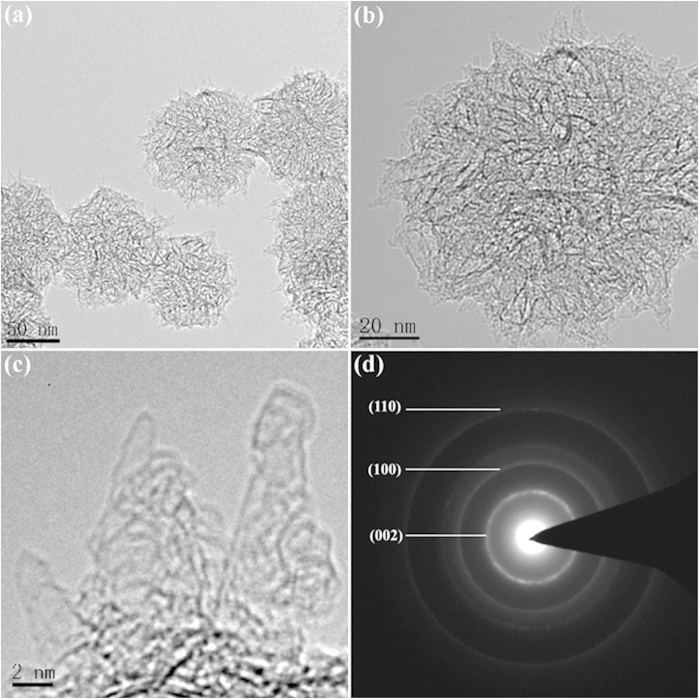
(**a**) TEM image of the CNHs, (**b**) high magnification TEM image of the CNHs, (**c**) locally enlarged image of the horn-shaped tips of the CNHs, and (**d**) SAED pattern of the CNHs.

Further insight into the morphology and microstructure of CNHs and FeF3·0.33H2O@CNHs nanocomposite were obtained by using a transmission electron microscope (TEM). As shown in Fig. 3a, the CNHs assemblies are formed by aggregation of spherical bundles with 80–100 nm in diameter. The detailed morphology of the “dahlia-like” nanostructure is shown in Fig. 3b,c. As shown, the entire structure of the 3D hierarchical architecture is constructed with thousands of closed tubes which have horn-shaped tips, the diameter of 2–5 nm, tubule length of 40–50 nm and cone angle of approximately 20°. These tubes connect to each other through the center to form 3D hierarchical “dahlia-like” structure. Furthermore, the selected are an electron diffraction (SAED) analysis (Fig. 3d) corresponding to CNHs give a set of diffraction rings, which can be clearly assigned to the diffractions of the (002), (101) and (110) planes, respectively.

**Figure 4:**
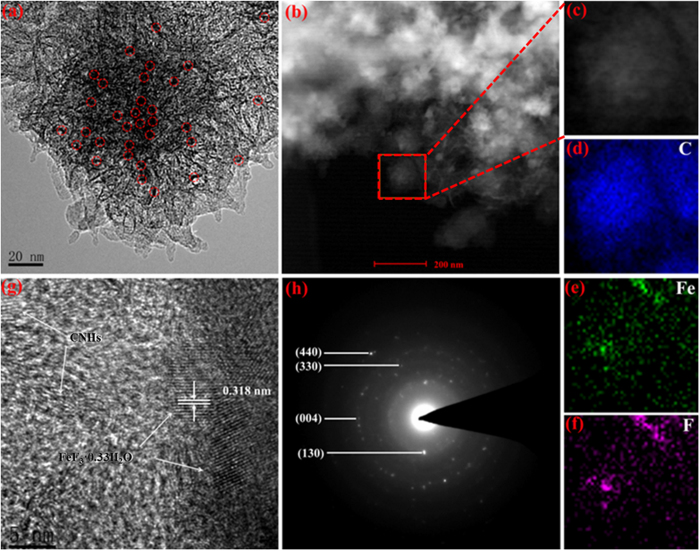
TEM, HAADF-STEM and elemental mapping of FeF_3_·0.33H_2_O@CNHs nanocomposite. (**a**) TEM image. (**b**) Typical STEM image (**c**) STEM image taken from the square region marked in (**b**). (**d**–**f**) Corresponding elemental mapping images of (**d**) C, (**e**) Fe, and (**f**) F. (**g**) High resolution TEM images. (**h**) SAED pattern.

Figure 4 shows the TEM image of the FeF_3_·0.33H_2_O@CNHs nanocomposite. As shown in Fig. 4a, FeF_3_·0.33H_2_O nanoparticles (labeled by red circles) with a size of ~5 nm are anchored in the 3D hierarchical “dahlia-like” structure which serves as the conductive matrix, after the impregnation and crystallization at 80 °C. Further evidence for the presence of Fe, C, and F components in the FeF_3_·0.33H_2_O@CNHs nanocompositeis were identified by High Angle Angular Dark Field-Scanning Transmission Electron Microscopy (HAADF-STEM) and elemental mapping (Fig. 3b–f). The elemental mapping images of Fe and F clearly demonstrate the FeF_3_·0.33H_2_O nanoparticles are homogeneous distribution in the 3D hierarchical “dahlia-like” CNHs, which insures intimate contact between the two components. As shown in Fig. 4g, the HRTEM image displays FeF_3_·0.33H_2_O nanoparticles strongly anchored on the CNHs, demonstrating the good contact between the CNHs and the FeF_3_·0.33H_2_O nanoparticles. The (040) plane of HTB-FeF_3_·0.33H_2_O can be clearly identified from the interlayer spacing of 0.318 nm. The selected area electron diffraction analysis (SAED) of the nanocomposite confirms the polycrystalline structure of FeF_3_·0.33H_2_O nanoparticles.

**Figure 5:**
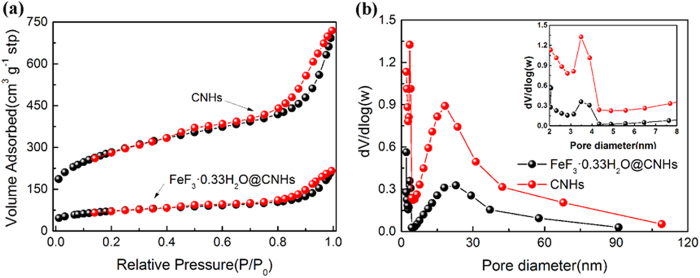
(**a**) Nitrogen sorption isotherms and (**b**) corresponding pore size distribution of CNHs and FeF_3_·0.33H_2_O@CNHs nanocomposite, Inset: an enlarged scale at low pore width.

The Brunauer-Emmett-Teller (BET) specific surface area and pore size distribution of the CNHs and FeF_3_·0.33H_2_O@CNHs nanocomposite were compared by nitrogen isothermal adsorption analysis. The nitrogen adsorption-desorption isotherms show that both samples were found to be of type IV isotherm with a clear H1 hysteresis loop (Fig. 5a), which was typical characteristics of mesoporous materials. After the impregnation and crystallization, the BET surface area is found to decrease from 959.8 m^2^ g^−1^ for CNHs to 268.9 m^2^ g^−1^ for FeF_3_·0.33H_2_O@CNHs nanocomposite. As shown in Fig. 5b, both samples exhibit bimodal pore size distribution, with the pore width of 3.5 nm and 20 nm. These two groups of pores can be attributed to the inter-nanohorn pores and intra-nanohorn pores, respectively. The high BET specific surface area of the FeF_3_·0.33H_2_O@CNHs nanocomposite (268.9 m^2^ g^−1^) can provide more reaction sites and is beneficial for electrolyte access. What’s more, the unique mesoporosity and the 3D hierarchical structure can promote the diffusion of electrolyte ions.

**Figure 6:**
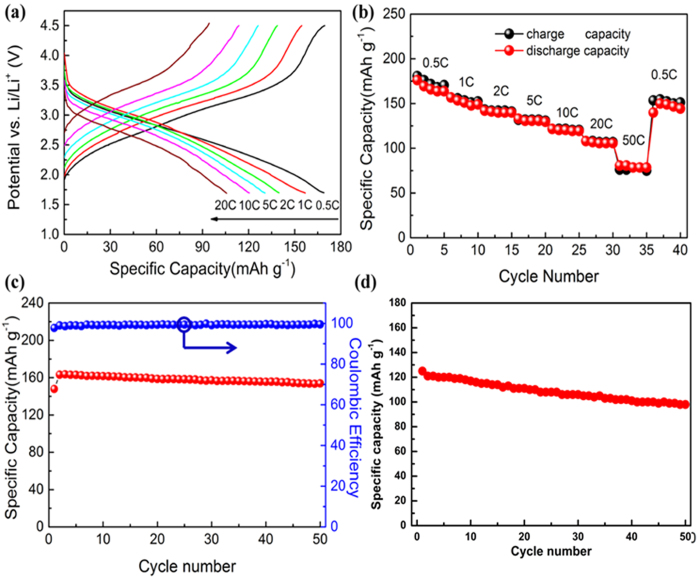
(**a**) Discharge profiles at different current rates for FeF_3_·0.33H_2_O@CNHs nanocomposite, (**b**) discharge capacity versus cycle number at different current rates for FeF_3_·0.33H_2_O@CNHs nanocomposite, (**c**,**d**) cyclic performance at 1 and 10 C rate for FeF_3_·0.33H_2_O@CNHs nanocomposite and the coulombic efficiency of the FeF_3_·0.33H_2_O@CNHs nanocomposite (1C).

To evaluate the electrochemical performance of the FeF_3_·0.33H_2_O@CNHs nanocomposite, we performed galvanostatic charge-discharge measurement based on the half-cell configuration. The typical discharge profiles of the compositesat different current rates (0.5 C, 1 C, 2 C, 5 C, 10 C and 20 C) are presented in Fig. 6a. During discharging, the profiles show sloped reaction plateaus, which stem from the Li^+^ insertion into the HTB-type FeF_3_·0.33H_2_O forming the single phase solid solution^15^,^19^,^35^. The reaction mechanisms have been demonstrated by Maier’s group as a simple Li-insertion reaction over a voltage range of 1.7–4.5 V^36^. As illustrated in Fig. 6a, the discharge capacities of the nanocomposite at 0.5 C, 1 C, 2 C, 5 C and 10 C are 169, 157, 140, 131 and 120 mAh g^−1^ , respectively. Even atthe high current rate of 20 C (4 A g^−1^), a capacity of 106 mAh g^−1^ can still be obtained.

As shown in Fig. 6b, the nanocomposite shows excellent ultrahigh rate cyclying performance. The 3D hierarchical nanocomposite exhibits excellent ratecapability with discharge capacities of 142 mAh g^−1^ at 2 C, 131 mAh g^−1^ at 5 C, and 108 mAh g^−1^ at 20 C. Notably, a discharge capacity of 81 mAh g^−1^ can still be obtained at a ultrahigh rate of 50 C. When the charge rate was set back to 0.5 C after 35 cycles, the reversible capacity of FeF_3_·0.33H_2_O nanocomposite returned to 150 mAh g^−1^. The greatly enhanced rate performance of the nanocomposite can be attributed to the tiny FeF_3_·0.33H_2_O nanocrystallites, conductive carbon network and unique hierarchical mesoporous structure, resulting in better electronic and ionic conduction throughout the electrode.

Figure 6c shows the cyclic performance of the FeF_3_·0.33H_2_O@CNHs nanocomposite at 1 C. The nanocomposite can still deliver a capacity of 154 mAh g^−1^ after 50 cycles with capacity retention over 95%, which corresponds to only 0.1% capacity loss percycle. The coulombic efficiency stayed constant at approximately 100% during the Li^+^ insertion/extraction process. Meanwhile, the cycling stability for high rate at 10 C was shown in Fig. 6d. After 50 cycles, the capacity of 98 mAh g^−1^ was retained. Evidently, the electrochemical performance demonstrated that the nanoparticles embedded in CNHs matrix form a stable structure on the microscale and that the electrochemical Li^+^ insertion/extraction process is highly reversible.

**Figure 7:**
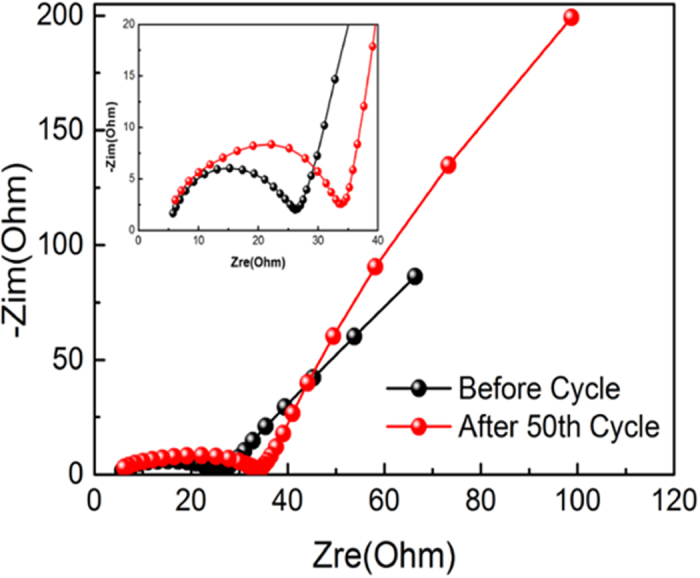
Nyquist plots of FeF_3_·0.33H_2_O@CNHs nanocomposite before and after 50 cycles at 1 C.

To explain the outstanding electrochemical performance of FeF_3_·0.33H_2_O@CNHs, the electrochemical impedance spectroscopy (EIS) was applied before cycling and after 50 cycles at 1 C (Fig. 7). The high-frequency semicircle corresponds to the charge-transfer resistance (*R*_*ct*_)^38^,^39^,^40^, the inclined line can beattributed to the diffusion process of lithium ion within the electrode. The values of *R*_*ct*_ increase slightly after 50 cycles, suggesting that the FeF_3_·0.33H_2_O@CNHs electrode owns high electron conductivity and a fast charge-transfer reaction for Li^+^ insertion/extraction.

The superior performance was determined to originate from the unique microstructure feature of the FeF_3_·0.33H_2_O@CNHs nanocomposite. Firstly, the nanocomposite electrodes possess well-defined, hierarchical mesoporous that facilitate the rapid Li^+^ transfer. The large specific surfacearea also provides a sufficient electrolyte/electrode contact area resulting in a reduction in the current density of unit surface area and an enhancement in the charge/dischargerate. Secondly, the growth and agglomeration of FeF_3_·0.33H_2_O nanoparticles confined between the nanohorns of the framework during crystallization has been intensively suppressed. Meanwhile, the carbon matrix provides stable support to the FeF_3_·0.33H_2_O nanoparticles. This robust structure will determinately enhance the cyclic stability of the nanocomposite. Finally, FeF_3_·0.33H_2_O nanoparticles are connected directly to the carbon matrix, constructing a superior conductive network that allows for enhancement of the electron conductivity and efficient charge transport of the composite. All those above would undoubtedly boost the electron and Li^+^ transfer in the 3D mesoporous electrodes, resulting in greatly enhanced Li^+^ storage properties.

## Conclusion

Herein, an advanced architectural design of the CNHs carried FeF_3_·0.33H_2_O nanocomposites (FeF_3_·0.33H_2_O@CNHs) are constructed for the first time through a facile solution-based approach. The CNHs play dual roles in the superior architecture, the one is to build up the conductive matrix which provides a charge transfer express way to FeF_3_·0.33H_2_O, another is to disperse and support the FeF_3_·0.33H_2_O nanoparticles as an “elastic confinement” carrier. By combining the high electron conductivity, confined nanosized FeF_3_·0.33H_2_O (~5 nm), hierarchical mesopores CNHs and the “elastic confinement” support, the FeF_3_·0.33H_2_O@CNHs nanocomposite demonstrates excellent ultrahigh rate capability and good cycling properties. Therefore, FeF_3_·0.33H_2_O@CNHs holds promise as a good LIBs cathode material for high power density battery application.

## Methods

### Synthesis of FeF_3_·0.33H_2_O@CNHs Nanocomposite

The CNHs was prepared according to the method previously reported^19^. The as-prepared CNHs powder (150 mg) was dispersed in concentrated HNO_3_ (150 mL, 68%, Sinopharm Chemical Reagent Co., Ltd) solution and stirred for 72 hour at room temperature for purification and better dispersion. In a typical process, 15 mg CNHs were fully dispersed in alcohol (100 mL) by a powerful ultrasonic treatment. After 1 hour ultrasonic treatment, Fe(NO_3_)_3_·9H_2_O (1 g, 99.99%, pursued from Crystal Pure Industrial Co., Ltd. without further purification) was added to the above solution with vigorous stirring for half hour. Then the green fluoride source [Bmim][BF_4_] (10 mL, 99%, pursued from Centre for Green Chemistry and Catalysis, LICP, CAS.) was added to the above solution with vigorous stirring for 1 hour. Then, the solution was continuously stirred at 80 °C for 6 hours. The resulting product was washed repeatedly with acetone and dried by freeze drying.

### Materials Characterization

The crystal structure of CNHs and the nanocomposites were characterized by X-ray diffraction (PANalytical X’Pert PRO, monochromated Cu Kα radiation 40 mA, 40 kV) andmicro-Raman spectroscope (HR800, 632.8 nm laser). Transmission electron microscopy (TEM), high-resolution transmission electron microscopy (HRTEM), and selected-area electron diffraction (SAED) measurements were obtained on a FEI Tecnai F20 microscope operated at 200 kV equipped with energy dispersive X-ray spectrometer (EDX). Scanning electron microscopy (Hitachi, SU8010) was applied to characterize the morphology of the materials. Brunauer-Emmett-Teller (BET) surface area and N_2_ adsorption/desorption isotherms were measured by using an ASAP 2020 (Micromeritics). The carbon content in the nanocomposite was measured by a Carbon-Sulfur elements Determinator (Elementar Vario EL).

### Electrochemical Characterizations

The cathode electrode was prepared by mixing the active material, carbon black and polyvinylidene fluoride in N-methyl pyrrolidinone with a weight ratio of 8: 1: 1. The slurry was coated onto a cleaned aluminum foil by a doctor-balding method and dried at 80 °C for 8 hours under vacuum. Then, the electrodes were cut into discs (15 mm) and dried at 80 °C for 6 hours. The mass loading of the active material was about 1.3 mg cm^−2^. Coin cells (CR2025) were assembled in an argon-filled glove-box (Mbraun, H_2_O < 0.1 ppm, O_2_ < 1 ppm) using metallic lithium as the counter/reference electrode, porous polypropylene films (two pieces, Celgard 2400) as the separator and LiPF_6_ (1 M) in ethylene carbonate, diethyl carbonate and ethyl methyl carbonate (1:1:1 in vol) as an electrolyte. Charge/discharge measurements were performed at various current rates (1 C is equivalent to 200 mA g^−1^) within a voltage window of 1.7–4.5 V (vs Li^+^/Li) (during the aobve potential window, the CNHs have not capacity, it acts as 3D conductive network and supporter) using a Neware battery test system. EIS measurements were performed using a PARSTAT 2273 advanced electrochemical system over the frequency range between 1 MHz and 100 mHz with the amplitude of an ac signal of 10 mV.

## Additional Information

**How to cite this article**: Fan, L. *et al.* Carbon Nanohorns Carried Iron Fluoride Nanocomposite with ultrahigh rate lithium ion storage properties. *Sci. Rep.* **5**, 12154; doi: 10.1038/srep12154 (2015).

## Supplementary Material

Supplementary Information
