# Supplementary material for: Carbon Nanohorns Carried Iron Fluoride Nanocomposite with ultrahigh rate lithium ion storage properties
Source: Sci Rep. 2015 Jul 15;5:12154. doi: 10.1038/srep12154 (PMC4502412; doi:10.1038/srep12154)
Supplement: Supplementary Information [file srep12154-s1.doc]

**Supplementary information**

**Carbon Nanohorns Carried Iron Fluoride Nanocomposite with** **ultrahigh rate lithium ion** **storage properties**

Lishuang Fan^1+^, Bingjiang Li^1+^, Naiqing Zhang^2,3*^, Kening Sun^2,3*^

^1^Department of Chemistry, Harbin Institute of Technology, Harbin, China, ^2^State Key Laboratory of Urban Water Resource and Environment, Harbin Institute of Technology, Harbin, China, ^3^Academy of Fundamental and Interdisciplinary Sciences, Harbin Institute of Technology, Harbin, China

^*^Correspondence to: [znqmww@163.com](mailto:znqmww@163.com) (N.Q. Z) or [keningsunhit@126.com](mailto:keningsunhit@126.com) (K.N.S)

^+^These authors contributed equally to this work.


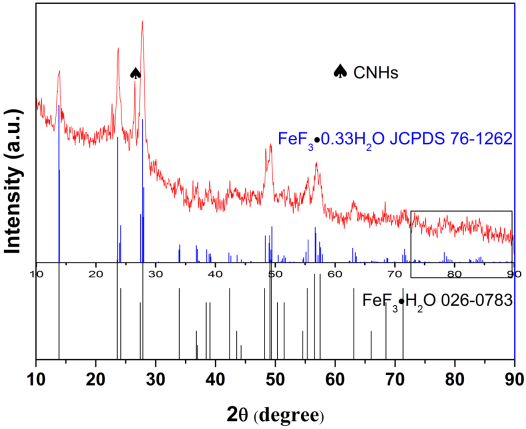


Figure S1 The X-ray diffraction of the FeF_3_·0.33H_2_O@CNHs nanocomposite.


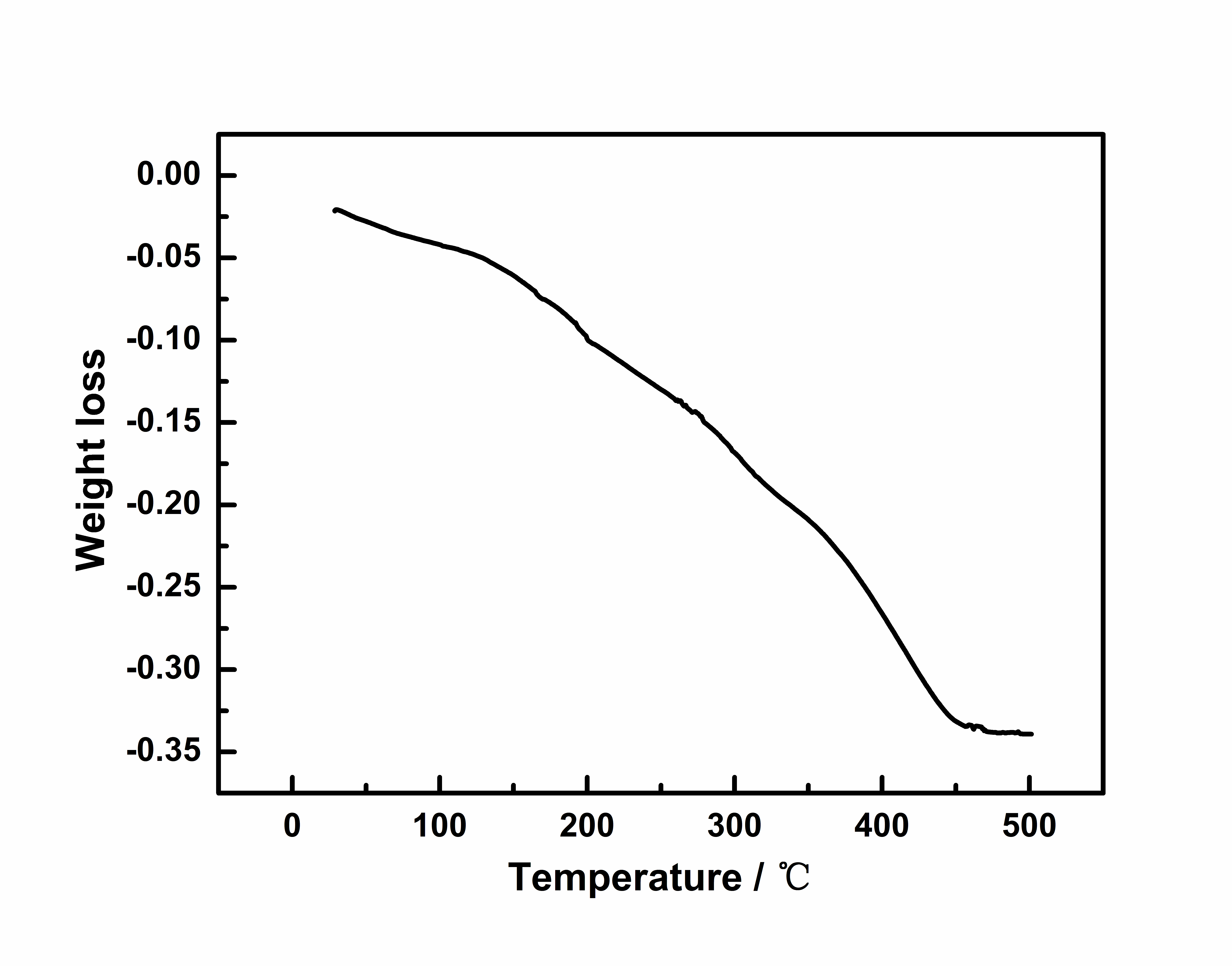


Figure S2 TG curve of FeF_3_•0.33H_2_O.
